# Assessment of the Photosynthetic Response of Potato Plants Inoculated with *Rhizoctonia solani* and Treated with Flesh-Colored Potato Extracts Nanoencapsulated with Solid Lipid Nanoparticles

**DOI:** 10.3390/plants14020156

**Published:** 2025-01-07

**Authors:** Sheina Rivas, Paola Fincheira, Felipe González, Christian Santander, Sebastián Meier, Cledir Santos, Boris Contreras, Antonieta Ruiz

**Affiliations:** 1 Programa de Doctorado en Ciencias de Recursos Naturales, Facultad de Ingeniería y Ciencias, Universidad de La Frontera, Temuco 4811230, Chile; 2 Departamento de Ciencias Químicas y Recursos Naturales, Scientific and Technological Bioresource Nucleus BIOREN-UFRO, Universidad de La Frontera, Temuco 4811230, Chile; 3 Laboratorio de Nanobiotecnología Ambiental, Centro de Excelencia en Investigación Biotecnológica Aplicada al Medio Ambiente (CIBAMA), Facultad de Ingeniería y Ciencias, Universidad de La Frontera, Av. Francisco Salazar 01145, Temuco 4811230, Chile; 4 Programa de Doctorado en Ciencias Mención Biología Celular y Molecular Aplicada, Facultad de Ciencias Agropecuarias y Medioambiente, Universidad de La Frontera, Temuco 4811230, Chile; 5 Instituto de Investigaciones Agropecuarias, INIA Carillanca, Casilla Postal 929, Temuco 4880815, Chile; 6 Escuela de Agronomía, Facultad de Ciencias, Ingeniería y Tecnología, Universidad Mayor, Campus Alemania, Temuco 4801143, Chile; 7 Novaseed Ltd.a., Loteo Pozo de Ripio s/n, Parque Ivian II, Puerto Varas 5550000, Chile; 8 Papas Arcoiris Ltd.a., Loteo Pozo de Ripio s/n, Parque Ivian II, Puerto Varas 5550000, Chile

**Keywords:** *Solanum tuberosum*, nanoencapsulation, chlorophylls, photosynthetic parameters

## Abstract

Potato has great nutritional and economic importance in agriculture. However, *Rhizoctonia solani* represents a significant risk, reducing the yield and quality of potato production. Flesh-colored potato (FCP) extracts show in vitro inhibitory effects against *R. solani*, although environmental factors may reduce their stability. Solid lipid nanoparticles (SNLs) offer a solution by encapsulating these compounds, preventing degradation, and improving delivery, positioning solid lipid nanoparticles as a promising technology for sustainable extract application. A greenhouse potato assay at two phenological stages under *R. solani* inoculation was used to evaluate the photosynthetic response (photosynthetic parameters and pigments) to two doses of the nanoencapsulated extract (SNL + FCP). During inoculation and commercial fungicide application, stomatal conductance, the photosynthetic rate, and the internal CO_2_ concentration increased compared with those of the non-inoculated control (NT), whereas the nanoencapsulated extract maintained levels similar to those of the NT, suggesting the possible regulation of the photosynthetic defense system. In terms of photosynthetic pigments, SLN + FCP maintained chlorophyll concentrations, unlike those in inoculated plants, which significantly decreased. Component analysis revealed that a lower dose primarily increased chlorophyll B synthesis, whereas a higher dose increased chlorophyll A compared with the inoculated control. These findings suggest an improved response from SLN + FCP to commercial fungicides, particularly with respect to photosynthetic pigments. However, further research is needed, and the results indicate promising potential for the eco-friendly control of phytopathogenic fungi in agriculture.

## 1. Introduction

The potato (*Solanum tuberosum* L.) is a crop that has earned a significant position as a raw material in food industry after maize, wheat, and rice in terms of human consumption [1,2]. Potato crops hold significant nutritional and economic value in Chile’s agricultural industry; this importance stems from the rich nutritional content of potatoes, which provides essential carbohydrates, vitamins, and minerals, making it a staple food for millions of people globally [3,4]. According to the Food and Agriculture Organization (FAO), potatoes play an increasingly significant role in food security, especially in developing regions. In Chile, potatoes rank fifth in terms of production, contributing 1,024,511.41 tons to the country’s food supply [2]. However, soil-borne phytopathogens represent a significant risk to potato production, reducing the yield and quality [5]. In alfalfa crops, it has been reported that, in warm temperatures, around 30 °C, and high humidity, dormant sclerotia of *Rhizoctonia solani* become activated, initiating mycelium growth [6]. This mycelium forms infection cushions, facilitating the penetration of the plant created by young lateral roots. Mycelium then progresses into the cortex region, which induces decay, leading to the sloughing off of cortex tissues [6]. As the infection advances, visible symptoms begin to appear in the plants [6]. In potato, black scurf is a disease caused by *R. solani*, which affects sprouts and can also cause damage by creating lesions in stolons and stems during early growth stages [7,8]. Additionally, after being harvested, sclerotia can develop on the epidermis of tubers, leading to further losses [9]. Currently, commercial fungicides are commonly used to protect potato crops despite their long-term toxicity to human health, wildlife, and the environment [10,11]. Flesh-colored potato (FCP) extracts have shown in vitro inhibitory activity against *R. solani* [12]. FCP is also a rich source of anthocyanins and hydroxycinnamic acid derivatives (HCADs) [13]. Nevertheless, environmental temperature and light exposure decrease the stability of polyphenols and coloration of extracts, which are decisive in the degradation or interconversion of phenolic compounds [14,15]. Accordingly, innovative technologies must be developed that allow their controlled release and protection against stresses from the environment.

In recent years, nanocarrier systems have been developed to increase the effectiveness of formulations applied in agriculture, minimizing potential impacts on the environment and human health [16,17,18]. Nanoencapsulation is a promising approach for loading a bioactive compound into a capsule. In particular, solid lipid nanoparticles (SLNs) (size: 50–1000 nm) composed of a solid lipid matrix encased by surfactants have been developed as an innovative technology. The lipids used for SLN preparation are usually phenological lipids that include glycerides, sterols, partial glycides, fatty acids, and waxes. To increase the stability of nanoparticles, all types of surfactants (neutral, ionic, and non-ionic) can be used, with the combination of more than one emulsifier helping to prevent particle agglomeration [19,20]. Furthermore, solid lipid nanoparticle materials are low-cost and eco-friendly alternatives that decrease environmental toxicity, allowing easy large-scale production, high efficacy, enhanced stability and solubility, protection, and the controlled release of the active compound [21]. Solid lipid nanoparticles can be used to reduce the requirement of significant concentrations of active agents, minimizing their losses due to leaching, degradation, and volatilization [22]. Therefore, it is a good option to encapsulate and preserve the stability of compounds, such as polyphenols as anthocyanins, the ones presented in flesh-colored potato extract, due to their easily degradation in an aqueous solution without a layer (lipids in these nanoformulations) to protect it [15].

Environmental challenges associated with conventional fungicides’ trace element toxicity highlight the need for innovative solutions in crop management [23,24]. Unlike traditional treatments, nanoformulations provide superior stability and reduce environmental impact by minimizing nutrient losses and ensuring targeted application. This aligns with the needs presented for sustainable alternatives to current agricultural practices [25]. Recent studies underline the potential of nanofertilizers to enhance agricultural sustainability. These formulations can improve nutrient use efficiency, decrease the accumulation of harmful substances in the environment (toxic trace elements from conventional fungicides application), and ensure the delivery of active agents precisely to target areas [25].

Furthermore, chemical fungicides also impact the photosynthetic processes of potato crops [26]. Photosynthesis is a process in plants that provides a nutrient supply and energy for various phenological and metabolic processes [27]. Photosynthetic parameters, such as the net photosynthesis rate (*A*), stomatal conductance (gs), and internal concentration of CO_2_ (Ci), play key roles in crop productivity and are closely related to yield productivity [28,29,30]. Nevertheless, this process can be disrupted by a range of complex abiotic and biotic factors, including both pathogens and fungicides [27,31]. Furthermore, while synthetic fungicides are used in potato crops to protect against pathogens, their application can lead to trace element toxicity, which poses risks to human health and the environment and potentially impacts the metabolic functions of plants, including nutrient uptake, antioxidant defenses, and changes in photosynthetic parameters [26,32]. The implementation of nanotechnology aims to preserve this FCP extract from degradation while providing a more ecofriendly solution for plant protection against phytopathogens. Considering these factors, formulations of solid lipid nanoparticles containing flesh-colored potato extract (FCP) have potential antifungal activity against *R. solani*, a pathogen of *S. tuberosum* crops. The objective of this study was to assess the effects of flesh-colored potato extracts nanoencapsulated with solid lipid nanoparticles on the photosynthetic performance of *S. tuberosum* leaves and compare their effects to those of commercial fungicides under *R. solani* inoculation.

## 2. Results

### 2.1. Physicochemical Characterization of Solid Lipid Nanoparticles Loaded with Flesh-Colored Potato Extract

**Figure 1:**
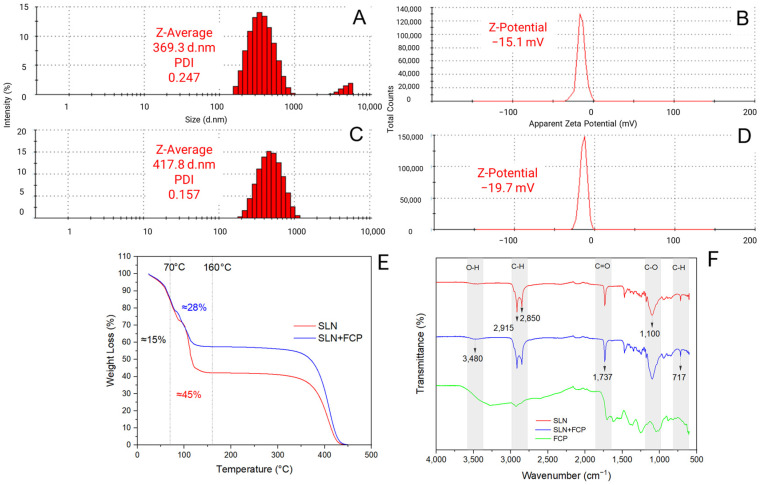
Physicochemical characterization of SLN loaded with FCP extract. (**A**) Size distribution of SLN + FCP; (**B**) zeta potential of SLN + FCP; (**C**) size distribution of SLN; (**D**) zeta potential of SLN; (**E**) thermogravimetric analysis (TGA); (**F**) Fourier transform infrared spectroscopy analysis (FTIR). SLNs: solid lipid nanoparticles, FCP: flesh-colored potato, SLN + FCP: solid lipid nanoparticle loaded with flesh-colored potato, SLN: formulation without FCP extract.

The formulation of solid lipid nanoparticles without FCP extract (SLNs) had a hydrodynamic size of 466.9 ± 0.5 nm and a polydispersity index (PDI) of 0.37 ± 0.04. Compared with the nanoencapsulated extract (SLN + FCP), the formulation exhibited a favorable size distribution (Figure 1A,C). The formulation and nanoencapsulated extract had Z-average values of 417.8 and 369.3 nm, respectively. The incorporation of liquid extract slightly increased the polydispersity index for the nanoencapsulated extract (0.247) compared with the formulation (0.157). Zeta potential (ZP) results for the nanoencapsulated extract and the formulations indicate negative values of −15.1 and −19.7 mV, respectively (Figure 1B,D). The results of thermogravimetric analysis (TGA) (Figure 1E) show that the mass loss decreases by approximately 15% in both samples as the temperature increases to 70 °C. As the temperature is further increased to 350 °C, the nanoencapsulated extract retains 57% of its original mass and the formulation of 40%. Fourier transform infrared (FTIR) spectra of the analyzed samples (SLN, SLN + FCP, and FCP) exhibited distinct absorption peaks at 2915, 2850, and 1737 cm^−1^, respectively. The same absorption peaks were observed at 3480, 1100, and 717 cm^−1^ for the formulation and nanoencapsulated extract. Importantly, FCP exhibited distinct peaks compared with the formulation and nanoencapsulated extract in the absorption interval between 2000 and 500 cm^−1^ (Figure 1F). The encapsulation efficiency (EE) of the nanoencapsulated extract (at 20 mg L^−1^ FCP extract) for total phenols (280 nm) was 99.33%, that for hydroxycinnamic acid derivatives (320 nm) it was 99.57%, and that for anthocyanins (520 nm) was 83.33%. Calibration curves: 280 nm: R^2^ = 0.9993; 320 nm: R^2^ = 0.9986; and 520 nm: R^2^ = 0.9749.

### 2.2. Evaluation of Photosynthetic Response

#### 2.2.1. Photosynthetic Parameters

**Figure 2:**
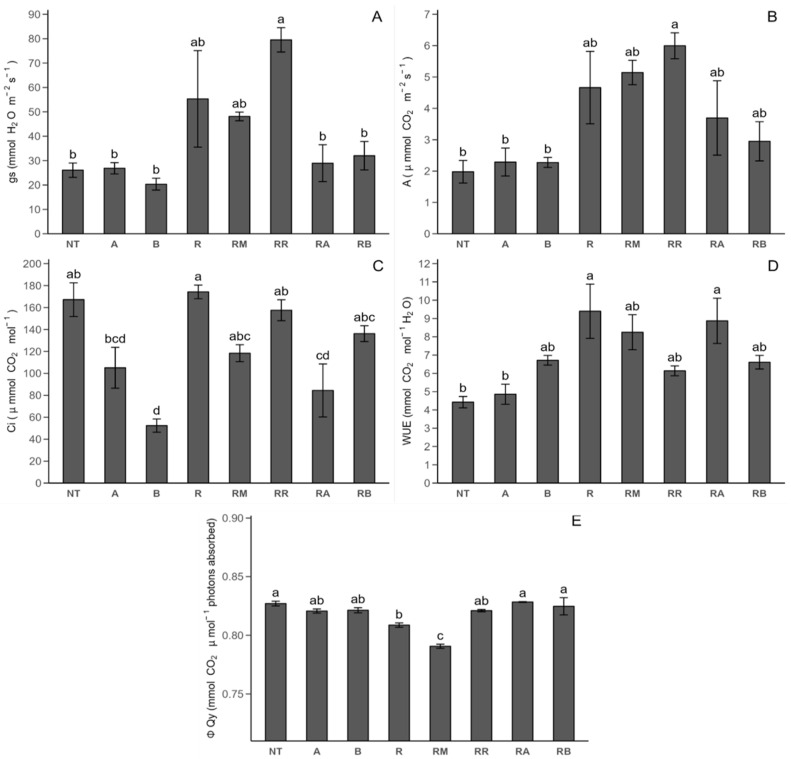
Photosynthetic parameter response of Stage 1 under different treatments with and without *R. solani* inoculation on potato crop leaves: (**A**) stomatal conductance (gs); (**B**) photosynthesis rate (*A*); (**C**) internal concentration of CO_2_ (Ci); (**D**) water use efficiency (WUE); (**E**) quantum yield of PSII (Qy). SLNs: solid lipid nanoparticles, FCP: flesh-colored potato, SLN + FCP: solid lipid nanoparticle loaded with flesh-colored potato, NT: *R. solani*-non-inoculated control and potato seed only, A: *R. solani*-non-inoculated control with SLN + FCP Dose A (20 mg L^−1^), B: *R. solani*-non-inoculated control with SLN + FCP Dose B (40 mg L^−1^), R: *R. solani*-inoculated control only, RM: *R. solani*-inoculated control with MONCUT fungicide, RR: *R. solani*-inoculated control with ReflectXtra fungicide, RA: *R. solani*-inoculated control with SLN + FCP (20 mg L^−1^), and RB: *R. solani*-inoculated control with SLN + FCP (40 mg L^−1^). Different letters indicate significant differences according to Tukey’s multiple range test (*p* < 0.05).

The initial photosynthetic parameters are related to Stage 1 (peak growth of potato plants) measurements (Figure 2). With respect to the photosynthetic response through stomatal conductance (gs: mmol H_2_O m^−2^ s^−1^) (Figure 2A), there was no significant difference between the *R. solani*-non-inoculated (NT) and *R. solani*-inoculated (R) controls. On the other hand, *R. solani* inoculated with ReflectXtra treatment (RR) increased the gs concentration by 204.91% compared with that of the NT control and SLN + FCP treatments (*R. solani*-non-inoculated control with SLN + FCP Dose A (A), *R. solani*-non-inoculated control with SLN + FCP Dose B (B), *R. solani*-inoculated control with SLN + FCP Dose A (RA), and *R. solani* inoculated control with SLN + FCP Dose B (RB)). In terms of the photosynthetic rate (*A*: μmol CO_2_ m^−2^ s^−1^) (Figure 2B), only the *R. solani*-inoculated control with ReflectXtra treatment resulted in a significant increase compared with that of the *R. solani*-non-inoculated control, but was not significantly different from that of the *R. solani*-inoculated controls. The internal concentration of CO_2_ (Ci: μmol mol^−1^) (Figure 2C) *R. solani*-non-inoculated control with SLN + FCP Dose B treatment resulted in a decrease in the internal concentration of CO_2_ compared with that of both controls. However, *R. solani*-inoculated with SLN + FCP Dose A presented a decrease of 49.49% compared with that of the non-inoculated control, suggesting a dose-dependent effect. In terms of the water use efficiency (WUE: mmol CO_2_ mol^−1^ H_2_O) (Figure 2D), there was a significant increase of 112.26% in this parameter for the *R. solani*-inoculated control compared with the non-inoculated control. Compared with the plants in the *R. solani*-non-inoculated control, the *R. solani*-inoculated plants in the SLN + FCP Dose A treatment group presented a significant increase in water use efficiency. Upon evaluating the fluorescence response through the quantum yield of PSII (QY-PSII: mmol CO_2_ μmol^−1^ absorbed photons) (Figure 2E), it was apparent that there was a decrease of 2.21% in the *R. solani*-inoculated control compared with the *R. solani*-non-inoculated control. Notably, only the *R. solani*-inoculated control with MONCUT (RM) treatment significantly differed from both the control and the *R. solani*-non-inoculated control with SLN + FCP Dose A, *R. solani*-non-inoculated control with SLN + FCP Dose B, *R. solani*-inoculated control with ReflectXtra treatment, *R. solani*-inoculated control with SLN + FCP Dose A, and *R. solani*-inoculated control with SLN + FCP Dose B treatments, all of which resulted in a decrease in the quantum yield of PSII for *R. solani*-inoculated control with MONCUT treatment. However, no significant differences were detected between the ReflectXtra and SLN + FCP treatments in terms of this parameter.

**Figure 3:**
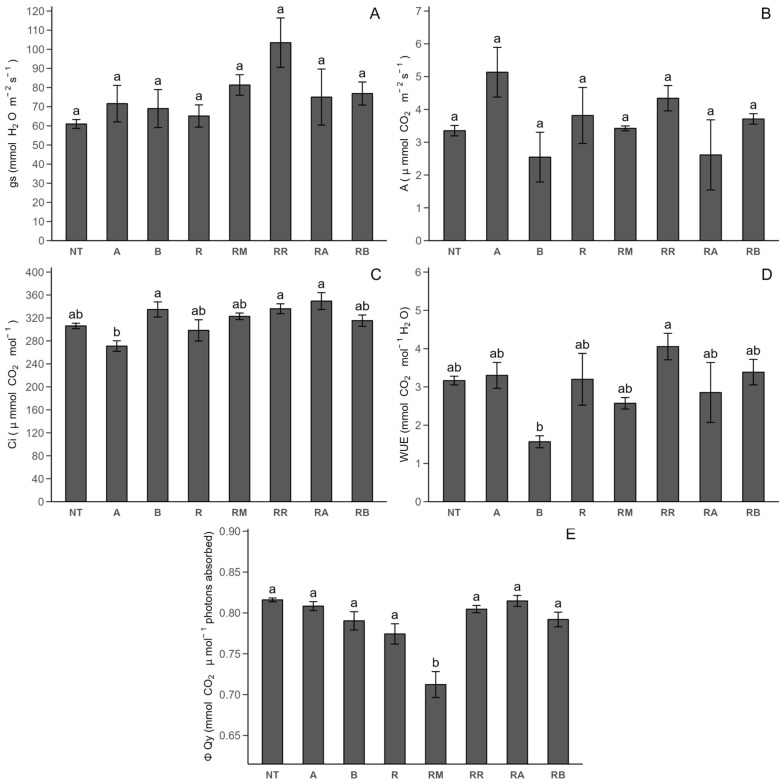
Photosynthetic parameter response of Stage 2 under different treatments with and without *R. solani* inoculation on potato crop leaves: (**A**) stomatal conductance (gs); (**B**) photosynthesis rate (*A*); (**C**) internal concentration of CO_2_ (Ci); (**D**) water use efficiency (WUE); (**E**) quantum yield of PSII (Qy). SLNs: solid lipid nanoparticles, FCP: flesh-colored potato, SLN + FCP: solid lipid nanoparticle loaded with flesh-colored potato, NT: *R. solani*-non-inoculated control and potato seed only, A: *R. solani*-non-inoculated control with SLN + FCP Dose A (20 mg L^−1^), B: *R. solani*-non-inoculated control with SLN + FCP Dose B (40 mg L^−1^), R: *R. solani*-inoculated control only, RM: *R. solani*-inoculated control with MONCUT fungicide, RR: *R. solani*-inoculated control with ReflectXtra fungicide, RA: *R. solani*-inoculated control with SLN + FCP (20 mg L^−1^), and RB: *R. solani*-inoculated control with SLN + FCP (40 mg L^−1^). Different letters indicate significant differences according to Tukey’s multiple range test (*p* < 0.05).

The previously mentioned photosynthetic parameters were measured again during Stage 2 (after flowering, close to senescence) (Figure 3). During Stage 2, we observed variations compared to Stage 1 outcomes. Regarding the stomatal conductance parameter (Figure 3A) and photosynthetic rate (Figure 3B), no significant differences were observed among the treatments. Regarding the internal concentration of CO_2_ (Figure 3C), there were no significant differences across most treatments, except for an increase in the internal concentration of CO_2_ in the *R. solani*-non-inoculated control with SLN + FCP Dose B treatment compared to its counterpart with Dose A. Concerning water use efficiency (Figure 3D), a similar pattern was observed as the internal concentration of CO_2_ results, except for an increase of 158.82% in the water use efficiency observed in the *R. solani*-inoculated control with ReflectXtra treatment compared to the *R. solani*-non-inoculated control with SLN + FCP Dose B treatment. Lastly, for the quantum yield of PSII (Figure 3E), no significant differences were noted among treatments, except for the *R. solani*-inoculated control with MONCUT treatment, which had a highly significant decrease in the quantum yield of PSII compared to other treatments.

#### 2.2.2. Photosynthetic Pigments

**Figure 4:**
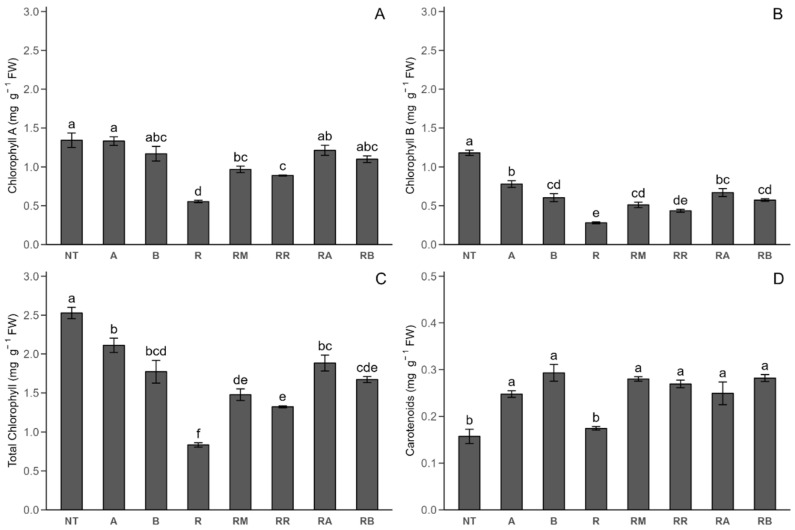
Photosynthetic pigment response of Stage 1 under different treatments with and without *R. solani* inoculation on potato crop leaves. (**A**) Chlorophyll A (ChA); (**B**) chlorophyll B (ChB); (**C**) total chlorophyll (ChTT); (**D**) carotenoid (Car). SLNs: solid lipid nanoparticles, FCP: flesh-colored potato, SLN + FCP: solid lipid nanoparticle loaded with flesh-colored potato, NT: *R. solani*-non-inoculated control and potato seed only, A: *R. solani*-non-inoculated control with SLN + FCP Dose A (20 mg L^−1^), B: *R. solani*-non-inoculated control with SLN + FCP Dose B (40 mg L^−1^), R: *R. solani*-inoculated control only, RM: *R. solani*-inoculated control with MONCUT fungicide, RR: *R. solani*-inoculated control with ReflectXtra fungicide, RA: *R. solani*-inoculated control with SLN + FCP (20 mg L^−1^), and RB: *R. solani*-inoculated control with SLN + FCP (40 mg L^−1^). Different letters indicate significant differences according to Tukey’s multiple range test (*p* < 0.05).

The initial presentation focuses on the results obtained from Stage 1 measurements of photosynthetic pigments (Figure 4). The chlorophyll A (ChlA) (Figure 4A) concentration did not significantly differ between the *R. solani*-inoculated control with SLN + FCP Dose A or B treatments and the non-inoculated control. However, a 58.73% decrease in the chlorophyll A concentration was observed in the *R. solani*-inoculated plants compared with the non-inoculated control plants. Compared with that in the *R. solani*-non-inoculated control, the concentration of chlorophyll A in the MONCUT treatment decreased (27.99%) and that in the *R. solani*-inoculated control increased (74.50%). The same trend was observed for the *R. solani*-inoculated control with ReflectXtra. Additionally, compared with the *R. solani*-inoculated control, the *R. solani*-inoculated control with SLN + FCP Dose A and *R. solani*-inoculated control with SLN + FCP Dose B increased the chlorophyll A concentration (109.07 and 98.37%, respectively). Chlorophyll B (ChlB) (Figure 4B) has lower concentrations than chlorophyll A. Compared with the *R. solani*-non-inoculated controls, *Rhizoctonia* inoculation reduces chlorophyll B synthesis. Compared with the *R. solani*-non-inoculated control, the *R. solani*-inoculated SLN + FCP treatments decreased the chlorophyll B concentration but increased the chlorophyll B concentration (139.46% Dose A and 104.90% Dose B) compared with the *R. solani*-inoculated control. A similar trend to that of chlorophyll B was observed for the total chlorophyll (ChlTT) (Figure 4C) concentration. In the presence of *R. solani*, nanoencapsulated extract application increased total chlorophyll synthesis, resulting in an important increase (Dose A, 125.91%; Dose B, 100.56%) compared with that of the *R solani*-inoculated control. For the carotenoid concentration (car) (Figure 4D) at this phenological stage, there was a significant increase in the carotenoid concentration for the *R. solani*-non-inoculated control with SLN + FCP Dose A, *R. solani*-non-inoculated control with SLN + FCP Dose B, *R. solani*-inoculated control with MONCUT treatment, *R. solani*-inoculated control with ReflectXtra treatment, *R. solani*-inoculated control with SLN + FCP Dose A, and *R. solani*-inoculated control with SLN + FCP Dose B, compared with both the *R. solani*-non-inoculated and *R. solani*-inoculated controls.

**Figure 5:**
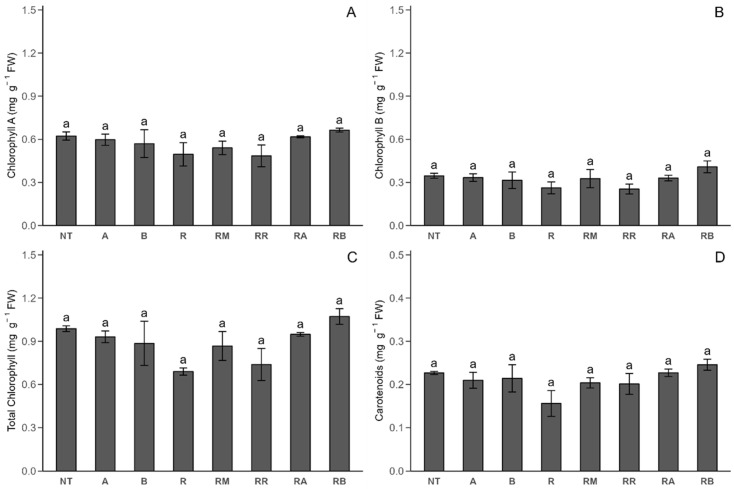
Photosynthetic pigment response of Stage 2 under different treatments with and without *R. solani* inoculation on potato crop leaves. (**A**) Chlorophyll A (ChA); (**B**) chlorophyll B (ChB); (**C**) total chlorophyll (ChTT); (**D**) carotenoid (Car). SLNs: solid lipid nanoparticles, FCP: flesh-colored potato, SLN + FCP: solid lipid nanoparticle loaded with flesh-colored potato, NT: *R. solani*-non-inoculated control and potato seed only, A: *R. solani*-non-inoculated control with SLN + FCP Dose A (20 mg L^−1^), B: *R. solani*-non-inoculated control with SLN + FCP Dose B (40 mg L^−1^), R: *R. solani*-inoculated control only, RM: *R. solani*-inoculated control with MONCUT fungicide, RR: *R. solani*-inoculated control with ReflectXtra fungicide, RA: *R. solani*-inoculated control with SLN + FCP (20 mg L^−1^), and RB: *R. solani*-inoculated control with SLN + FCP (40 mg L^−1^). Different letters indicate significant differences according to Tukey’s multiple range test (*p* < 0.05).

In Stage 2 (Figure 5), no significant differences were found in the chlorophyll A, chlorophyll B, total chlorophyll, or carotenoid concentrations across the treatments. However, a slight decrease in both total chlorophyll and carotenoids was detected in the *R. solani*-inoculated control.

### 2.3. Multivariate Analysis

**Figure 6:**
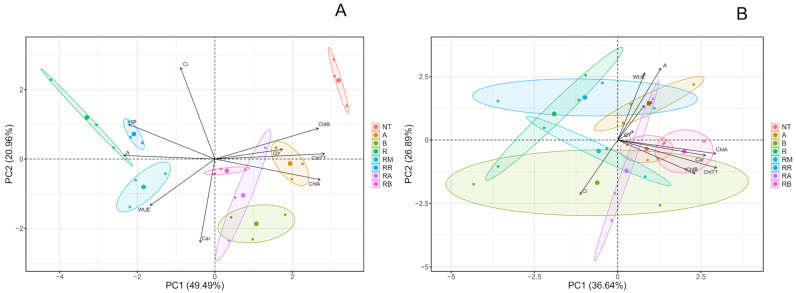
Multivariate analysis for the general photosynthetic response at Stage 1 and Stage 2 under different treatments with and without *R. solani* inoculation on potato crop leaves. (**A**) Stage 1; (**B**) Stage 2. The percentage values in parentheses indicate the variation explained by each principal component (PC). The plot and circles show the distribution of the experimental samples according to the PCs. SLNs: solid lipid nanoparticles, FCP: flesh-colored potato, SLN + FCP: solid lipid nanoparticle loaded with flesh-colored potato, NT: *R. solani*-non-inoculated control and potato seed only, A: *R. solani*-non-inoculated control with SLN + FCP Dose A (20 mg L^−1^), B: *R. solani*-non-inoculated control with SLN + FCP Dose B (40 mg L^−1^), R: *R. solani*-inoculated control only, RM: *R. solani*-inoculated control with MONCUT fungicide, RR: *R. solani*-inoculated control with ReflectXtra fungicide, RA: *R. solani*-inoculated control with SLN + FCP (20 mg L^−1^), and RB: *R. solani*-inoculated control with SLN + FCP (40 mg L^−1^). ChlA: chlorophyll A, ChlB: chlorophyll B, ChlTT: total chlorophyll, Car: carotenoid, QY: quantum yield of PSII, Ci: internal concentration of CO_2_, gs: stomatal conductance, A: photosynthetic rate, and WUE: water use efficiency.

A principal component analysis (PCA) (Figure 6, Tables S1–S4) was performed to analyze the eight treatments used in the Stage 1 measurement (Figure 6A). Both the photosynthetic pigments and the photosynthetic parameters were considered. The results indicate that the response is closely associated with photosynthetic pigments, particularly in the *R. solani*-non-inoculated control, non-inoculated control with SLN + FCP Dose A treatment, and inoculated control with SLN + FCP Dose A treatment. Notably, the *R. solani*-non-inoculated control with SLN + FCP Dose A and *R. solani*-inoculated control with SLN + FCP Dose A treatments had a significant effect on the synthesis of total chlorophyll, as well as fluorescence, through the quantum yield of PSII and, most importantly, the synthesis of chlorophyll B. In addition, a similar response in terms of the chlorophyll B content was detected in the *R. solani*-non-inoculated control. Second, the synthesis of chlorophyll A is associated with both *R. solani*-inoculated and non-inoculated plants that received SLN + FCP Dose B treatments. A correlation was also noted between *R. solani*-non-inoculated and inoculated plants that received SLN + FCP Dose A treatments. Furthermore, photosynthetic parameters, such as the internal concentration of CO_2_, stomatal conductance, and photosynthetic rate, were observed. The *R. solani*-inoculated control with ReflectXtra fungicide treatment resulted in a response through stomatal conductance, whereas the *R. solani*-inoculated control with the control resulted in a photosynthetic rate. Conversely, the internal concentration of CO_2_ did not appear relevant in treatments or the overall response. Finally, the use of the *R. solani*-inoculated control with MONCUT fungicide is linked to water use efficiency parameters, although it lacks a statistically significant effect on the total photosynthetic response, similar to that of carotenoids. However, there was a close and positive relationship between chlorophylls and the quantum yield of PSII with Doses A and B of the flesh-colored potato extract encapsulated by solid lipid nanoparticles. Despite this, the response through photosynthetic parameters was not highly significant, although there was a stronger association between commercial fungicide treatments and *R. solani*-inoculated treatments. In Stage 2 (Figure 6B), clear effects on the studied parameters were not evident. In this scenario, stomatal conductance, the photosynthetic rate, and water use efficiency could provide insights into the photosynthetic response, particularly in the *R. solani*-non-inoculated control with SLN + FCP in the Dose A treatment. These results suggest that both the *R. solani*-non-inoculated control and the *R. solani*-inoculated SLN + FCP Dose B treatment could be explained by the response involving photosynthetic pigments, including chlorophyll A, chlorophyll B, total chlorophyll, and carotenoids. Finally, clarity regarding other treatments (*R. solani*-non-inoculated control with SLN + FCP Dose B, *R. solani*-inoculated control, *R. solani*-inoculated control with MONCUT treatment, *R. solani*-inoculated control with ReflectXtra treatment, and *R. solani*-inoculated control with SLN + FCP Dose A) and their relationships with photosynthetic parameters and pigments is lacking. Intriguingly, the observed results appear to be significantly different from the Stage 1 outcome in Figure 6A.

## 3. Discussion

The zeta potentials of the nanoencapsulated extract, −15.1 mV, and the formulation, −19.7 mV, fall within the optimal range (−30 to 30 mV) for the colloidal system stability of particles. This study revealed that, compared with the nanoencapsulated extract, the majority of the formulation particles presented a more favorable size distribution. This difference could be attributed to the incorporation of the liquid extract, as it may contain other interfering substances. Notably, the polydispersity index by homogeneous-sized NPs was successfully formulated (PDI = 0.247 for SLN + FCP and 0.157 for SLN), where 0.2 is considered highly satisfactory [33]. The thermogravimetric analysis results reveal a weight loss of only 5% at a temperature of 50 °C for both the formulation and nanoencapsulated extract. It is crucial to avoid material and mass loss due to exposure to high temperatures, especially high temperatures during the summer season. Fourier transform infrared analysis of the mid-IR spectrum (400–4000 cm^−1^) was conducted following the method outlined by Nandiyanto et al. [34]. The peak at 3480 cm^−1^ was observed in all three samples, suggesting the presence of a characteristic O-H vibration band in the structure of glyceryl tristearate, as well as in key groups of the flesh-colored potato extract, such as anthocyanins and hydroxycinnamic acid derivates. Absorption peaks in the range of C–H bonds (2915 and 2850 cm^−1^) are indicative of aliphatic compounds, as are found in the hydrocarbon chains of glyceryl tristearate. This analysis suggests that the flesh-colored potato extract does not overlay the surface of the solid lipid nanoparticle formulation, supporting its efficient encapsulation [35]. Consequently, specific functional groups of FCP are not prominently displayed in the nanoencapsulated extract compared with the formulation. The results obtained for the encapsulation efficiency of the nanoencapsulated extract are very promising, showing a percentage greater than 80% for all measured wavelengths; this is particularly remarkable, as it surpasses the threshold of 70% EE [36,37,38].

In Stage 1, the correlation between the stomatal conductance and a heightened photosynthetic rate aligns with the literature, emphasizing that increased stomatal opening leads to elevated CO_2_ consumption and a reduced internal concentration of CO_2_ within the leaf [39]. Marek et al. [40] and Holz et al. [41] reported significant increases in stomatal conductance levels with fungicide use in tomato and barley, respectively, which is consistent with our findings. However, fungicide application can variably impact stomatal conductance, with some studies showing increases and others showing decreases compared with those in the untreated controls, reflecting a range of responses in different crops. Gómez et al. [12] reported similar stomatal conductance values in uninoculated potato leaves, but reported significant differences with the FCP extract. Cayún et al. [26] reported no significant differences with MONCUT or ReflectXtra, along with a decrease in stomatal conductance. The photosynthetic rate, as reported in potato [12], decreased by 92.8% in *R. solani*-inoculated plants compared with non-inoculated controls, contrary to our study. In tomato and barley [40,41], a decline in photosynthetic rate values was observed for inoculated plants, with fungicides significantly increasing the photosynthetic rate value in both inoculated and non-inoculated plants. For the internal concentration of CO_2_, no significant differences were detected between the controls and potato leaves [12], with values higher than those reported in this study. In tomato, there was a significant decrease in the internal concentration of CO_2_ under *A. solani* inoculation compared with that in non-inoculated plants [40]. In contrast, a significant decrease in the water use efficiency was observed in potato and tomato leaves of plants inoculated with pathogens compared with non-inoculated plants [12,40]. Additionally, a previous report did not find significant differences between the MONCUT and ReflectXtra treatments and the control without inoculation. Other reports [12,40] revealed a significant decrease in the quantum yield of PSII in the inoculated control compared with the non-inoculated control, which is consistent with the results presented here. In the potato [26], ReflectXtra significantly decreased the quantum yield of the PSII value compared with the non-inoculated control, whereas in the tomato [40], the opposite effect was observed with different fungicides. Compared with the *R. solani*-non-inoculated and *R. solani*-inoculated controls, the addition of different treatments did not influence the plants significantly when they were in the flowering stage. The variability in the response of photosynthetic parameters, including stomatal conductance, photosynthetic rate, internal concentration of CO_2_, water use efficiency, and quantum yield of PSII, is evident across diverse crops and in the presence of various fungicides, with no discernible pattern [41,42]. Specific factors, such as crop type and environmental conditions, may influence this variability [43,44]. Therefore, additional research is needed to explore the impact on photosynthetic parameters in potato crops treated with commercial fungicides versus a potentially eco-friendly option, such as FCP extract encapsulated by solid lipid nanoparticles, given the scarcity of the related literature.

In Stage 1, the total chlorophyll concentration in the *R. solani*-non-inoculated control was similar to that in the potato leaf treatment [26], while the chlorophyll A and chlorophyll B values were lower than those reported in this study. However, no significant differences were detected between the control and commercial fungicides in that study, which contrasts with our findings where higher values were obtained. In tomato leaves, fungicide application did not significantly affect chlorophyll A, chlorophyll B, total chlorophyll, or carotenoid concentrations compared with those in the control [45]. However, for pathogen inoculation, these concentrations significantly decreased compared with those of the non-inoculated control [40,46], which aligns with the results of this research. In various crops, such as cassava and grapevine, the contents of chlorophylls and carotenoids are reduced in the presence of commercial fungicides [47,48,49]. However, in different crops (tomato and cassava) under pathogen inoculation, fungicides significantly increase the concentration of photosynthetic pigments, which is also observed in the *R. solani*-inoculated control with SLN + FCP in the A and B treatments [40,41,49]. While commercial fungicides tend to increase photosynthetic pigments in the presence of biotic stress, the results of the *R. solani*-inoculated control with SLN + FCP Dose A and *R. solani*-inoculated control with SLN + FCP Dose B treatments showed a greater increase in the chlorophyll content than the effects of fungicides. Similar findings were reported by Iwaniuk and Lozowicka [50], where there was a significant increase in carotenoid concentrations due to fungicide application compared with those of the controls, which is consistent with our results. Additionally, Stage 2 presented lower photosynthetic pigment values than Stage 1, which could be attributed to the relationship between the water content and chlorophyll content [51], as it is nearing senescence periods, leading to a loss of leaf turgor [52], making water acquisition challenging. Finally, the results of the *R. solani*-inoculated control with SLN + FCP Dose A and *R. solani*-inoculated control with SLN + FCP Dose B treatments indicate a potential protective mechanism against stress, as reflected by increased concentrations of photosynthetic pigments, which is consistent with the findings for wheat and grapevine [49,53,54].

Principal component analysis (PCA) was conducted to examine the responses of the eight treatments under study in terms of their photosynthetic parameters and photosynthetic pigments. In Stage 1, a secondary outcome of the component analysis, the fungicides’ MONCUT, and ReflectXtra affected the photosynthetic parameters in this study, an occurrence also noted with *Vitis vinifera* extract [12]. These findings suggest a growth-promoting effect rather than a plant defense mechanism against *R. solani* in potato plants. In Stage 2, an initial response in the photosynthetic parameters was observed, which, although unclear in terms of which treatments induced it, might indicate the potential regulation of the photosynthetic apparatus as it approaches the senescence period. Furthermore, in other studies involving different crops, the application of systemic fungicides induced a defense mechanism through the synthesis of chlorophylls and carotenoids [40,49,55,56]. However, our results indicate a close relationship in this response when Doses A and B of the nanoencapsulated extract are applied; this could be explained by the fact that flesh-colored potato extract encapsulated by solid lipid nanoparticles, which is primarily composed of phenolic compounds, is associated with the phenological and biochemical responses of plants to stress [57,58]. Additionally, a correlation between the antioxidant activity and FCP extract was reported in potato plants inoculated with *R. solani* [12]. Finally, the *R. solani*-inoculated control with SLN + FCP Dose A and *R. solani*-inoculated control with SLN + FCP Dose B treatments increased the synthesis of photosynthetic pigments under *Rhizoctonia* inoculation, suggesting potential protection against the pathogen by promoting plant defense mechanisms. Considering that nanoencapsulated extract Dose A is more related to chlorophyll A synthesis and that nanoencapsulated extract Dose B affects chlorophyll B synthesis, a dose–response relationship should be further investigated.

The beneficial impact of nanoencapsulated extract on stages 1 and 2 of potato growth could be explained by the adequate direct contact and interaction with the seed and *R. solani*. Solid lipid nanoparticles have been proposed to encapsulate antifungal extracts to improve their stability, protection, and water solubility against environmental conditions. The immobilization of flesh-colored potato extract inside solid lipids protects it against oxidative, photochemical, and chemical degradation under real application conditions. Moreover, the highly stable colloidal solution, biodegradable nature, and nanoscale range of solid lipid nanoparticles enhance the capacity to penetrate target pests and maintain the efficiency of the antifungal activity of FCP. In this context, the solution of a nanoencapsulated extract irrigated in the surrounding substrate protects potatoes through the controlled release of phenols, anthocyanins, and hydroxycinnamic acids contained in the flesh-colored potatoes. The application of 5 mL of nanoencapsulated extract at 20 and 40 mg L^−1^ allowed exert protective action during stages 1 and 2 of the growth of the potatoes due to the sustainable release of adequate concentrations of bioactive compounds. The release of phenols, anthocyanins, and hydroxycinnamic acids from the formulation depends on the interaction behavior between the compounds and the lipid matrix with compounds. The retention and release of each compound depend on the stabilization inside the lipid matrix. Once the compounds present in the flesh-colored potato extract are released from the lipid phase of the nanoparticles, they can act on living organisms. The lipid nature of solid lipid nanoparticles allows their interaction with the cell membrane of potatoes and *R. solani*, allowing them to exert an antifungal action. The hydrophobic characteristics of the formulation allow their interaction with the cell membrane of fungi, altering its fluidity and the release of FCP into the cell. In general, natural extract controls fungi growth through (1) the inhibition of the synthesis of RNA, DNA, and protein; (2) the inhibition of cell wall formation and division; (3) the inhibition of efflux pumps and production of reactive oxygen species; and (4) the dysfunction of fungal mitochondria activity. Owing to the limited research on this topic under the studied conditions, this finding is crucial and could significantly contribute to the agricultural industry by reducing the use of toxic systemic fungicides, offering a more environmentally friendly option.

## 4. Materials and Methods

### 4.1. Reagents and Materials

Flesh-colored potatoes (CB2011-104 genotype) provided by Novaseed Ltd.a. (Puerto Varas, Chile) were used for the preparation of the FCP extract. The reagents’ glacial acetic acid, palmitic acid, lauric acid, stearic acid, hexane, methanol, monopotassium phosphate, urea, and acetone were obtained from Merck (Darmstadt, Germany). The reagents ethanol 95%, Tween 80, and Tween 20 were obtained from Winkler (Santiago, Chile). Glyceryl tristearate was acquired from Sigma–Aldrich (Darmstadt, Germany) and dichloromethane was acquired from Scharlau (Barcelona, España). Chemical fungicides were MONCUT, Hihon Nohyaku Co., Ltd. (Tokio, Japan) and ReflectXtra, Syngenta (Stirlingshire, UK).

### 4.2. Preparation of Flesh-Colored Potato Extract

Ground tubers (500 g) were homogenized with 1000 mL of 15% glacial acetic acid in ethanol, sonicated for 1 min, stirred for 30 min and centrifuged [14]. The FCP extract was rotary and resuspended in a 0.05% Tween 20 solution as the maintenance solvent [12]. This extract was previously characterized [14] and shown to contain high levels of anthocyanins and hydroxycinnamic acids. The flesh-colored potato extract contained a total of 202 mg L^−1^ anthocyanins and 383 mg L^−1^ HCADs. The identified anthocyanins were petunidin-caffeoylrutinoside-5-glucoside, petunidin-3-coumaroylrutinoside-5-glucoside, petunidin-3-feruloylrutinoside-5-glucoside, malvidin-3-coumaroylrutinoside-5-glucoside, and a petunidin derivative. The hydroxycinnamic acid derivates were identified as 5-caffeoylquinic acid and 4-caffeoylquinic acid.

### 4.3. Formulation of Solid Lipid Nanoparticles Loaded with Flesh-Colored Potato Extract

A solid lipid nanoparticle formulation was formulated via high-shear homogenization followed by ultrasonication. This method was conducted according to Fincheira et al. [37] with slight modifications [59,60]. For the aqueous phase, Tween 20 at 1.5% *v*/*v* was dissolved into 40 mL of distilled water at 70 °C. Meanwhile, the lipid phase was prepared by melting glyceryl tristearate at 5% *w*/*v* in 5 mL of hexane at the same temperature. FCP extract was added to the lipid phase to reach a final solution at 20 or 40 mg L^−1^ (minimum inhibitory concentration, MIC). Then, the lipid phase was gradually added to the aqueous phase and homogenized by the stirring speed (10,000 rpm, 5 min) using Ultraturrax OV5 (Velp^®^, Scientifica, Usmate Velate, Italy). The resulting solution was sonicated with an ultrasonic processor (Sonics and Materials, Newtown, CT, USA) for 5 min (5 s on/off, 35% amplitude) and cooled at 4 °C (vertical refrigerated cabinet, HYC-290, Haier^®^, Qingdao, China). The solid lipid nanoparticle sample loaded with flesh-colored potato extract was identified as SLN + FCP, and the formulation without the flesh-colored potato extract was identified as SLN. The compositions of the solid lipid nanoparticle formulations are shown in Table S5.

### 4.4. Physicochemical Characterization of Solid Lipid Nanoparticle Formulations

The hydrodynamic size (size measured as a particle in an aqueous suspension), polydispersity index, and zeta potential of the formulation and nanoencapsulated extract were determined via dynamic light scattering (DLS) via Zetasizer ZS90 equipment (Malvern Instruments, Malvern, UK). The samples were diluted with 0.01% deionized water. Measurements were performed in polystyrene/polystyrene cells (10 × 10 × 45 mm) at 25 °C at an angle of 90°. To determine the zeta potential, the samples were placed in an electrophoresis instrument (DTS1079 cells, Malvern, Zetasizer Nano series). Thermogravimetric analysis was performed through a Simultaneous Thermal Analyser (STA) 6000 (Perkin Elmer, Waltham, MA, USA). A 20 mg sample was heated in a nitrogen atmosphere from 25 °C to 500 °C at a rate of 15 °C per minute [37]. Additionally, Fourier transform infrared spectroscopy analysis was conducted with an Agilent Technologies Cary 630 FTIR spectrophotometer (Santa Clara, CA, USA) to generate spectra within the transmittance interval of 3600 to 600 cm^−1^. Prior to analysis, the samples were lyophilized [35]. To determine the encapsulation efficiency, a calibration curve was constructed using major compounds in the flesh-colored potato extract: hydroxycinnamic acid derivatives and anthocyanins. This was conducted considering a 20 mg L^−1^ concentration of flesh-colored potato extract. The formulation and nanoencapsulated extract mixture (30 mL each) were centrifuged (9000 rpm, 15 min), and the supernatant phases (aqueous and lipid) were separated and further diluted (500 mg/10 mL). The absorbance was measured at 280 nm for phenolic compounds, 320 nm for HCADs, and 520 nm for anthocyanins via a UV–Vis spectrophotometer. Encapsulation efficiency was calculated via the following formula:

(1) $$EE\left(\%\right)=\frac{C\left(total\right)-C(free)}{C(total)}\times 100$$

### 4.5. Greenhouse Application of the Nanoencapsulated Extract in Potato Plants Inoculated with Rhizoctonia solani

This study was conducted in an outdoor greenhouse affiliated with Departamento de Ciencias Químicas y Recursos Naturales of Universidad de La Frontera in southern Chile. The environmental conditions of the greenhouse varied with the seasons from September 2023 to January 2024, with temperatures ranging from 6 °C to 28 °C, alternating rainy and sunny days, and a relative humidity ranging from 60% to 80%. A Photosynthetic assessment was conducted at two phenological stages of potato plant growth: one during peak growth (Stage 1) and the other after flowering, close to senescence (Stage 2). For this in vivo study, the flesh-colored potato extract was nanoencapsulated with solid lipid nanoparticles (SLNs + FCP) and was utilized as a potential antifungal agent. Two different dosages were employed, with Dose A at 20 mg L^−1^ and Dose B at 40 mg L^−1^. The application consisted of administering 5 mL of each dose directly on the substrate before placing the potato seed, following indications of ReflectXtra fungicide, which was used as an example for the application of the nanoencapsulated extract. The experimental design followed a completely randomized layout with six replicates for each treatment. The *R. solani* strain (accession code: RGM 2705-INIA) was obtained from the National Culture Repository at the Instituto de Investigaciones Agropecuarias in Chile; it was cryopreserved at −80 °C using glycerol and mineral oil. For replication, the strain was incubated in liquid potato dextrosa media for 12 days at 28 °C, and an inoculation mixture with a concentration of 10^4^ mycelia was prepared. To plant the seeds, 11 L pots were filled with a peat-based medium made of 100% brown peat granules (less than 25 mm) with a pH of 4.5. After sowing, each seed was fertilized with 100 mL of KH_2_PO_4_ and urea solution [61], initially 24 h after planting and again on day 102. Irrigation began after the first fertilization and was adjusted daily based on weather conditions.

The following treatments were performed according to various factors: Treatment 1: non-inoculated treatment (NT); Treatment 2: SLN + FCP Dose A, 20 mg L^−1^ (A); Treatment 3: SLN + FCP Dose B, 40 mg L^−1^ (B); Treatment 4: *R. solani* inoculation (R); Treatment 5: *R. solani* inoculation and application of MONCUT (RM); Treatment 6: *R. solani* inoculation and application of ReflectXtra (RR); Treatment 7: *R. solani* inoculation and application of SLN + FCP Dose A, 20 mg L^−1^ (RA); and Treatment 8: *R. solani* inoculation and application of SLN + FCP Dose B, 40 mg L^−1^ (RB). Treatments with 5 mL of SLN + FCP were initially applied to the substrate surrounding the potato seed and pots were sealed. Each treatment had 6 replicates, resulting in n=48 (Table 1).

### 4.6. Photosynthetic Determination

The evaluation of photosynthetic and fluorescence characteristics was performed twice during the cultivation period to capture two different phenological stages of potato plants (Stage 1 and Stage 2). Data collection occurred on 21 November 2023 and 8 January 2024, and the data were aligned with specified periods. To collect the data, a Targas-1 system (PP Systems, Amesbury, MA, USA) was used to evaluate stomatal conductance (gs: mmol H_2_O m^−2^ s^−1^), the photosynthetic rate (*A*: μmol CO_2_ m^−2^ s^−1^), the internal concentration of CO_2_ (Ci: μmol mol^−1^), and water use efficiency (WUE: mmol CO_2_ mol^−1^ H_2_O). All measurements were conducted in vivo, 2 h after the initiation of the photoperiod, utilizing the second most-recent leaf. Fluorescence measurements were executed with a FluorPen system (Photon System Instrument, Drasov, Czech Republic) incorporating version 1.0 software, which enabled the determination of the quantum yield of photosystem II (QY-PSII: mmol CO_2_ μmol^−1^ absorbed photons).

### 4.7. Photosynthetic Pigment Contents in Leaves

Photosynthetic pigments, including total chlorophyll content, chlorophyll A, chlorophyll B, and carotenoids, were assessed in this study. Leaf harvests were conducted on the day following the measurement of photosynthetic parameters (22 November 2023 and 9 January 2024), and two sets of data were obtained. The evaluation of these pigments adhered to established methodologies by Lichtenthaler and Wellburn [62,63], with slight modifications.

### 4.8. Statistical Analysis

All figures and statistical analyses were conducted in R version 4.2.1. After confirming the normality and homoscedasticity of the data, the datasets were subjected to one-way analysis of variance (ANOVA). For variables that exhibited significant differences, means were compared via Tukey’s HSD multiple range test, with a significance level of *p* < 0.05 established for all cases. These analyses were performed via the R library “agricolae” v.1.3.5. Bar charts representing the means ± standard errors are presented, and significant differences are indicated by different letters between treatments. Additionally, the dataset was split by stage of development and standardized to ensure that all variables contributed equally to the analysis. This was achieved by scaling the data to unit variance and mean centering each variable (z-score normalization) and subjected to principal component analysis. The loadings matrix was computed to show the contribution of each variable to the principal components and the percentage of total variance explained by each principal component was calculated. The confidence ellipses (group means) by treatment were also generated using the packages “FactoMineR”, version 2.7, and “factoextra”, version 1.0.7.

## 5. Conclusions

The initial results of this study demonstrate the successful physicochemical characterization of the formulation “SLN” and the nanoencapsulated extract “SLN + FCP”, considering the essential nanoparticle characteristics. Compared with the *R. solani*-inoculated control, the nanoencapsulated extract treatment had promising effects on increasing chlorophyll levels. The identified dose-dependent responses and protective mechanisms warrant further exploration, emphasizing the potential of ecofriendly alternatives in agriculture. This study provides a foundation for reducing the reliance on chemical fungicides, highlighting that solid lipid nanoparticles offer a sustainable strategy for encapsulating flesh-colored potato extracts. This, in turn, enables its potential application in agricultural systems for the biocontrol of phytopathogenic fungi for sustainable and environmentally friendly agricultural practices.

## Figures and Tables

**Table 1:** Experimental design of study treatments, with detailed used/not used factors.

Name	Potato Seed	*R. solani*	Fungicide	SLN + FCP	Replicates
NT	Yes	No	No	No	6
A	Yes	No	No	Dose A	6
B	Yes	No	No	Dose B	6
R	Yes	Yes	No	No	6
RM	Yes	Yes	MONCUT	No	6
RR	Yes	Yes	ReflectXtra	No	6
RA	Yes	Yes	No	Dose A	6
RB	Yes	Yes	No	Dose B	6

## Data Availability

The data presented in this study are available upon request from the corresponding authors.

## Supplementary Materials

The following supporting information can be downloaded at: https://www.mdpi.com/article/10.3390/plants14020156/s1, Table S1: Results of the principal component analysis (PCA) showing the eigenvalues, percentage of variance explained by each principal component (PC), and the cumulative percentage of variance explained for the dataset of peak growth (Stage 1) in the development of *Solanum tuberosum*; Table S2: Results of the principal component analysis (PCA) showing the eigenvalues, percentage of variance explained by each principal component (PC), and the cumulative percentage of variance explained for the dataset of after flowering close to senescence in the development of *Solanum tuberosum* (Stage 2); Table S3: Contribution matrix of the principal component analysis (PCA) showing the contribution (%) of each variable to the principal components (PCs) for the dataset of peak growth (Stage 1) in the development of *Solanum tuberosum*. The values indicate the relative importance of each variable in defining the respective principal components; Table S4: Contribution matrix of the principal component analysis (PCA) showing the contribution (%) of each variable to the principal components (PCs) for the dataset of after flowering close to senescence development of *Solanum tuberosum* (Stage 2). The values indicate the relative importance of each variable in defining the respective principal components; Table S5: Constituents of formulations of solid lipid nanoparticles (SLNs).
