# Supplementary material for: Assessment of the Photosynthetic Response of Potato Plants Inoculated with Rhizoctonia solani and Treated with Flesh-Colored Potato Extracts Nanoencapsulated with Solid Lipid Nanoparticles
Source: Plants (Basel). 2025 Jan 7;14(2):156. doi: 10.3390/plants14020156 (PMC11768129; doi:10.3390/plants14020156)
Supplement: Supplementary file 1 [file plants-14-00156-s001.zip › plants-3368205-supplementary.pdf]

Supplementary Material.

"Assessment of the photosynthetic response of potato plants inoculated with *Rhizoctonia solani* and treated with flesh-coloured potato extracts nanoencapsulated with solid lipid nanoparticles"

Table S1: Results of the principal component analysis (PCA). showing the eigenvalues. percentage of variance explained by each principal component (PC). and the cumulative percentage of variance explained for the dataset of peak growth (stage 1) in the development of *Solanum tuberosum*.

Component	eigenvalue	percentage of variance	cumulative percentage of variance
PC1	4.4539	49.4883	49.4883
PC2	1.8866	20.9622	70.4505
PC3	1.019	11.322	81.7725
PC4	0.7368	8.1869	89.9594
PC5	0.6866	7.6290	97.5883
PC6	0.1596	1.7732	99.3615
PC7	0.0468	0.5196	99.8811
PC8	0.0107	0.1188	99.9999
PC9	0.0000	0.0001	100.0000

Table S2: Results of the principal component analysis (PCA). showing the eigenvalues. percentage of variance explained by each principal component (PC). and the cumulative percentage of variance explained for the dataset of after flowering. close to senescence in the development of *Solanum tuberosum* (Stage 2).

Component	eigenvalue	percentage of variance	cumulative percentage of variance
PC1	3.2976	36.6395	36.6395
PC2	2.4197	26.8850	63.5246
PC3	1.1367	12.6302	76.1548
PC4	1.0113	11.2362	87.3910
PC5	0.6521	7.2460	94.6371
PC6	0.3548	3.9423	98.5794
PC7	0.1072	1.1909	99.7703
PC8	0.0133	0.1479	99.9182
PC9	0.0074	0.0818	100.0000

Table S3: Contribution matrix of the principal component analysis (PCA). showing the contribution (%) of each variable to the principal components (PCs) for the dataset of peak growth (stage 1) in the development of *Solanum tuberosum*. The values indicate the relative importance of each variable in defining the respective principal components.

Variable	PC1	PC2	PC3	PC4	PC5	PC6	PC7	PC8	PC9
ChlTT	20.11918	0.13902	3.45872	8.04191	0.24450	3.15853	0.02737	0.40316	64.4076
ChlA	18.56847	2.10881	7.14517	4.33734	0.25583	3.09144	46.35805	0.47108	17.66382
ChlB	17.89557	4.67726	0.75589	11.10786	0.20554	2.47831	41.52597	3.42585	17.92773
Car	0.36538	34.4182	14.39205	10.9119	9.65228	24.11851	5.32348	0.81773	0.00047
Gs	12.46613	5.95994	30.25451	0.29118	2.05766	1.29019	1.68419	45.99616	0.00003
A	13.93550	0.06488	22.68394	10.48074	9.24028	0.54691	2.23935	40.80824	0.00015
Ci	1.98298	41.50616	0.90630	3.29402	2.90136	46.43893	2.13522	0.83488	0.00015
WUE	7.01001	10.66002	19.22331	10.76498	26.29862	18.8307	0.20933	7.00297	0.00006
QY	7.65679	0.46569	1.18010	40.77007	49.14392	0.04647	0.49704	0.23992	0.00000

Table S4: Contribution matrix of the principal component analysis (PCA). showing the contribution (%) of each variable to the principal components (PCs) for the dataset of after flowering. close to senescence development of *Solanum tuberosum (Stage 2)*. The values indicate the relative importance of each variable in defining the respective principal components.

Variable	PC1	PC2	PC3	PC4	PC5	PC6	PC7	PC8	PC9
ChlTT	25.70158	4.77232	0.00000	0.38964	2.50308	2.04477	1.85044	48.4169	14.32127
ChlA	25.28048	1.12517	0.42529	0.14329	5.87901	9.74064	55.72806	1.67729	0.00077
ChlB	15.73517	6.93401	0.50868	3.86377	35.23482	9.90763	0.21044	21.78918	5.81629
Car	20.22186	1.47047	0.06242	3.12201	35.34341	0.00002	31.60429	5.80765	2.36786
Gs	1.86001	7.3149	56.27068	8.04984	3.09155	5.31286	0.00287	4.68235	13.41494
A	4.89901	31.76118	0.46532	1.22363	2.80943	7.45923	3.02518	11.56857	36.78846
Ci	3.73565	18.2222	18.06443	20.35851	0.30770	5.17452	1.44859	5.97785	26.71057
WUE	1.92693	27.90823	0.03957	2.51637	3.33066	58.48971	5.60977	0.07394	0.10481
QY	0.63931	0.49151	24.1636	60.33294	11.50033	1.87063	0.52037	0.00627	0.47503

Table S5. Constituents of formulations of solid lipid nanoparticles (SLN).

**Formulation**	**Solid lipid 5 w/v%**	**Solvent**	**Surfactant 1.5 v/v%**	**Sonication time (min)**	**Homogenization time (min)**
SLN	Glyceryl tristearate	Dichloromethane	Tween 20	6	5
